# Vitamin D Deficiency among Blood Transfusion Dependent Beta Thalassemia Children Admitted to Tertiary Level Pediatric Hospital in Nepal: A Descriptive Cross-sectional Study

**DOI:** 10.31729/jnma.8779

**Published:** 2024-10-31

**Authors:** Anil Kumar Shrestha, Sangay Chultim Sherpa, Bindu Gyawali, Manisha Sharma, Santosh Adhikari, Suchitra Shrestha, Susan Bhattarai, Sagar Thapa, Devashish Sharma, Prajwal Paudel, Sushil Gyawali

**Affiliations:** 1 Department of Pediatrics, Kanti Children's Hospital, Maharajgunj, Kathmandu, Nepal; 2 Department of Medicine, Om Hospital and Research Center, Chabahil, Kathmandu, Nepal; 3 Curative Service Division, Ministry of Health and Population, Teku, Kathmandu, Nepal; 4 Nepalese army Institute of Health Science, Bhandarkhal, Kathmandu, Nepal; 5 Department of Pediatrics, Paropakar Maternity & Women's Hospital, Thapathali, Kathmandu, Nepal; 6 Department of Pediatric Surgery, Kanti Children's Hospital, Maharajgunj, Kathmandu, Nepal

**Keywords:** *beta thalassemia*, *blood transfusion*, *children*, *iron overload*, *Nepal*, *vitamin D deficiency*

## Abstract

**Introduction:**

Children with beta thalassemia are on regular blood transfusions, which could result in iron deposition in the liver causing decreased synthesis of Vitamin D-25OH. There are limited publications on the association of Vitamin D deficiency with blood transfusion-dependent thalassemia in the Nepalese population. This study aims to determine the prevalence of Vitamin D deficiency among blood transfusion-dependent beta-thalassemia patients.

**Methods:**

This was a descriptive cross-sectional study conducted among beta-thalassemia major patients under 15 years of age, receiving regular blood transfusion, from July 17, 2022, to July 16, 2023, after attaining ethical approval from Ethical Review Committee, (reference number 155). Data were collected using convenience sampling, and descriptive analyses were performed using Microsoft Excel and Statistical Package for Social Sciences (SPSS) 2024.

**Results:**

A total of 127 blood transfusion-dependent beta-thalassemia major patients were included in the study, of whom 82 (64.56%) were female. Among these patients, 104 (81.88%) were aged between 5 and 14 year. Among 127, 41 (32.28%) had Vitamin D insufficiency, and 31 (24.40%) had Vitamin D deficiency. There were 12 (9.44%) underweight children.

**Conclusions:**

Vitamin D deficiency was seen in more than half of the children with blood transfusion dependent beta thalassemia major.

## INTRODUCTION

Thalassemia is a common congenital disorder affecting 190 million carriers globally, with 240,000 infants born yearly.^1^ Among its clinical subtypes, beta-thalassemia major is the most severe form.^2,3^ It requires chronic blood transfusions, iron chelation, splenectomy, stem cell transplantation, and supportive treatment.^4,5^ Consequences of regular blood transfusions is associated with iron overload in the liver leading to decreased synthesis of Vitamin D-25OH. Beta-thalassemia is more prevalent in Southeast-Asia, including Nepal, where the lack of screening programs further masks the disease burden. Although precise prevalence data of Nepal is limited, the increasing number of diagnosed cases highlights its significant public health issue.^6,7^

Early identification of iron overload with Vitamin D supplementation is crucial to prevent growth and developmental delays. The study's main objective is to determine the prevalence of Vitamin D deficiency among blood-transfusion-dependent beta-thalassemia major children.

We aim to fill an important evidence gap in Nepal, as limited research has been conducted on this topic, thereby providing useful data to inform clinical practice and public health policies in Nepal.

## METHODS

This was a descriptive cross-sectional study conducted retrospectively among beta-thalassemia major patients receiving regular blood transfusions admitted to Thalassemia Unit, at a pediatric tertiary care hospital in Nepal for a period of one year from July 17, 2022 to July 16, 2023. Ethical approval was obtained from the Institutional Review Committee, Kanti Children Hospital (reference number 155). All patients within the study period and meeting inclusion criteria wre included in the study.

The study had inclusion criteria of children diagnosed with beta-thalassemia major aged 0-14 years who were on regular follow-up in the thalassemia unit, Kanti Children's Hospital, and receiving regular blood transfusions without comorbid conditions and with complete medial records. Exclusion criteria included children with comorbidities such as congenital heart disease, malignancy, chronic liver disease, epilepsy, or any other chronic diseases, other forms of thalassemia, as well as patients with incomplete medical records. In this retrospective study the data were obtained from hospital records maintained by medical record section including medical charts from the thalassemia unit and then were recorded on a predetermined proforma. Individual consent from the cases were not sought. These variables included age, gender, ethnicity, height, weight, vitamin D level, serum calcium level and serum ferritin level.

Serum Vitamin D level more than 30 ng/ml was taken as normal, value between 20-30 ng/ml was taken as insufficiency and value less than <20 ng/ml ws taken as deficiency; serum level of Vitamin D <30 ng/ml was termed as Vitamin D abnormality. Similarly, normal serum calcium level of 8-10 mg/dl and normal serum ferritin level of 30-150 pg/liter was considred as normal.^6,8^ The weight of a child was measured in kilograms and height in centimeters.

For assessment of malnutrition in the children, children were divided into two categories: below 5 years of age and 5 to 18 years of age. For the age below 5 years, height-for-age z-score more than 2 standard deviations below the WHO Child Growth Standard median was considered as stunting and weight-for-age z-score more than 2 standard deviations below the WHO Child Growth Standard deviation was considered as underweight.^7,9^ For the children 5 years to 18 years, height-for-age less than the 3^rd^ percentile was considered as stunting and weight-for-age less than the 3^rd^ percentile was considered as underweight.^8,10^

The variables from proforma were entered into a Microsoft Excel sheet. The statistical analysis was performed using IBM Statistical Package for Social Sciences (SPSS) Statistics version 24.

## RESULTS

A total of 136 medical charts were reviewed, out of which 127 meet the inclusion criteria. The nine excluded cases were; 4 alfa-thalassemia and 5 with incomplete medical records.

There were 45 (35.43%) male. There 41 (32.28%) were Janjatis in the population under study (Table 1).

**Table 1:** Socio-demographic representation of Transfusion-Dependent Beta Thalassemia Children (n=127).

Variables	n (%)
**Sex**	
Male	45 (35.43)
Female	82 (64.57)
**Age group**	
< 1 year	3 (2.36)
1 to 4 years	20 (15.75)
5 to 14 years	104 (81.89)
**Ethnicity**	
Bramhin/Chhetri	30 (23.62)
Dalit	29 (22.83)
Janjati	41 (32.28)
Madhesi	21 (16.54)
Others	6 (4.72)
Total Cases	127

The mean value of serum vitamin D level was 28.91±13.16 ng/dl. Vitamin D abnormalities (<30 ng/ml) were present in 75 (59.05%) cases. The average serum calcium level was 9.43±2.10 mg/dl with 125 (98.43%) cases had normal levels and 2 (1.57%) cases had lower levels. The average serum ferritin level was 4149.5±3008.2 pg/l, and 5 (3.94%) cases had normal and 122 (96.06%) cases with elevated levels.

The study showed Vitamin D deficiency (<20 ng/ml) in 34 (26.77%) cases, Vitamin D insufficiency (20 to <30) in 41 (32.28%) cases, and normal level (>30 ng/dl) in 52 (40.94%) cases.

Out of 34 vitamin D deficient cases, 1 (2.94%) case was in <1 year age group, 4 (11.76%) cases were in 1 to 4 years age group and 29 (85.29 %) cases were in 5 to 14 years age group. (Table 2).

**Table 2:** Vitamin D level according to age groups in Transfusion-Dependent Beta Thalassemia Children (n=75).

Age group	Classification	<1 year	1-4 year	5-14 year
	Deficiency (n=34)	1 (2.94%)	4 (11.76%)	29 (85.29%)
	Insufficiency (n=41)	0	3 (7.32%)	38 (92.68%)

**Figure 1:**
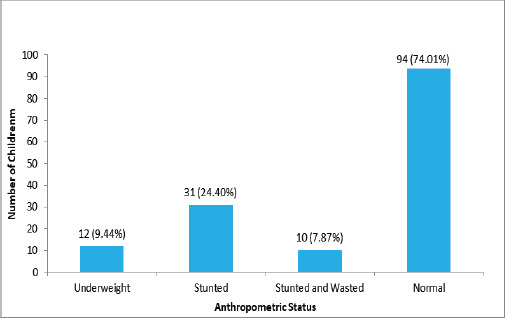
Nutritional Status of Transfusion-Dependent Beta Thalassemia Children (n=127) (According to WHO and IAP Growth Standards).

In the study population 31 (24.40%) of population were found to be stunted (Figure 1).

WHO: World Health Organization, IAP: Indian Acdemy of Pedritician

## DISCUSSION

Beta thalassemia is an inherited blood disorder characterized by reduced levels of functional hemoglobin. Beta thalassemia has three main forms minor, intermedia and major, which indicate the severity of the disease. Its severe form beta thalassemia major almost always requires regular blood transfusions lifelong and also ongoing medical care. Because of repeated blood transfusions individuals may develop excess levels of iron in the body. Impaired calcium homeostasis is thought to be a consequence of iron overload seen in multi-transfused thalassemia patients. Defective synthesis of 25 OH vitamin D has been described in these patients which negatively affect their bone metabolism. Beta thalassemia used to be a potential lethal disease previously, but advancement in therapies with optimized transfusion programs have improved the quality and life expectancy of these patients.^11^ Measurement of serum ferritin level is an easy and cost effective measure for starting iron-chelation therapy.^12^

The mean serum vitamin D level was 28.9±13.1 ng/ml. This level is higher as compared to the study done by Dhale S et al. in India (2019) which reported the mean vitamin D level of 16±8.75 ng/ml.^13^ The difference could be due to variations in dietary intake, sunlight exposure and the effectiveness of institutional practice of vitamin D supplementation in different regions. In our cross-sectional study, the data of the most recent laboratory values of the cases were included, irrespective of the previous laboratory values and supplementation of Vitamin D or iron chelating agents.

Vitamin D deficiency and insufficiency status were found in 26.77% and 32.28% of the cases, respectively. This high prevalence is consistent with the study by Dhale S et al. which reported 33% of patients being Vitamin D deficient and 44.4% being insufficient.^11^ Similarly Fung EB et al. (2011) reported that 73% of beta thalassemia patients were vitamin D deficient and 33% were insufficient.^14^ In addition, Vogiatzi MG et al reported that 12-8% cases had 25 vitamin D concentrations less than 27 nmol/l and 82% less than 75 nmol/l, regardless of the thalassemia syndrome.^15^ In other study by Napoli N et al,^8^ patients (10.1%) with thalassemia major had serum 25-OH-vitamin D less than 10.4 ng/ml and were considered with an absolute deficiency of vitamin D and the mean 25-OH-vitamin D was significantly (P<0.01) lower in those beta thalassemia major patients.^16^ Other studies have also reported lower vitamin D levels in beta thalassemia patients.^17,18,19^

The mean serum calcium level was 9.4±2.1 mg/dl with 98.42% of participants having normal calcium levels. This is consistent with the findings of Vogiatzi MG et al. (2009) who reported normal serum calcium levels in beta thalassemia patients despite varying degrees of vitamin D deficiency.^15^ Normal serum calcium levels suggest that the compensatory mechanisms might be at play, maintaining calcium homeostasis despite vitamin D insufficiency and also there could be oral calcium supplementation.

Similarly mean serum ferritin level was 4149.5±3008.2 μg/L with 96.06% of participants having elevated levels. Repeated blood transfusion leading to iron overload contributes to rise in serum ferritin level. Elevated levels of serum ferritin levels in blood transfusion dependent thalassemia patients were also reported by the similar studies done by Bhalodiya VR et al (2023) that reported mean serum ferritin level in 0-5 year age group of 1262 μg/l and in 6-10 years age group of 1963.44 μg/l, and by Dejkhamron P et al. (2018) that reported 50.8% of vitamin D deficient cases with more tendency of cardiac siderosis in vitamin D deficient cases, which emphasized the need for effective iron chelation therapy for the management of iron overload.^12,20^

The mean age of the children was 7 years and 8 months in our study with range of age from 9 months to 14 years. Most children (82%) were between 5 to 14 years of age, and 18% were <5 year old. A study by Fahim FM, et al. (2013) found similar age distribution in thalassemia major patients, showing the age-related increase in endocrine complications such as vitamin D deficiency.^21^ Similar result has been shown in study conducted by De Sanctis V et al (2013).^1^

We observed the male to female ratio of 1:1.8, with 64.56% of participants being female which contradicts with the study done by Manolopoulos PP et al (2021) which reported higher prevalence of beta thalassemia in males. Also studies done by Bhalodiya VR et al. (2023), Chahkandi T et al. (2006) and Hamayun T et al.(2017) have reported similar predominance in males.^12,17,22^. About 10% cases were underweight and 24.40% cases were stunted in our study. These findings contrast with a study by Fahim et al. (2013) that have showed a higher prevalence of malnutrition among thalassemia major patients. The study observed 49 % short stature and 47% underweight cases in thalassemia major patients who received multiple blood transfusions.^21^

The strength of our study is that it was conducted in one of the tertiary level pediatric hospital of government of Nepal with its Thalassemia unit providing service to a large number of patients. The result in this study might have represented the blood transfusion dependent thalassemia patients throughout the nation that have potentially limited the selection bias. This study being the first of its kind in Nepal, can provide valuable data on the burden of beta thalassemia patients, their morbidity status, and the prevalence of Vitamin D abnormalities and iron levels. Therefore it will be useful for relevant stakeholders and can also guide physicians and healthcare providers in managing the disease effectively.

As this study was retrospective and cross-sectional in nature in which the data included the most recent laboratory reports of some of the patients with normal vitamin D levels who had vitamin D deficiency in their previous reports, which could have been corrected with vitamin D supplementation. If we were able to include those previous reports in the study, the real scenario of Vitamin D deficiency status could be different in comparison to the result from the recent reports only. This was the first limitation of our study. The another limitation is that we didn't consider the age at onset of thalassemia and the start of blood transfusion and also the total number of blood transfusion(s) the patients received. It would have been better if we were able to compare the association between the numbers of blood transfusion with the abnormalities in serum vitamin D level and ferritin level. Similarly, we didn't categorize the patients according to the geographical location, which could have reflected the prevalence of beta-thalassemia in different regions of the country.

Therefore prospective studies are recommended to overcome the shortcomings faced by our study. It is also important to emphasize that treatment of beta thalassemia patients with aggressive nutritional support including fortified cereals, fortified milk and supplementation with vitamin D.^10^

## CONCLUSIONS

More than half of the children with beta thalassemia major receiving multiple blood transfusions had Vitamin D deficiency. Almost all the patient in had iron overload while in most of the patients, serum calcium was normal. Nearly one third of the patient had normal nutritional status.
